# Coprophagy in moose: A first observation

**DOI:** 10.1002/ece3.9757

**Published:** 2023-01-19

**Authors:** Robert Spitzer, Cecilia Åström, Annika Felton, Monica Eriksson, Erling L. Meisingset, Erling J. Solberg, Christer M. Rolandsen

**Affiliations:** ^1^ Department of Wildlife, Fish and Environmental Studies Swedish University of Agricultural Sciences Umeå Sweden; ^2^ Southern Swedish Forest Research Centre Swedish University of Agricultural Sciences Alnarp Sweden; ^3^ Department of Forestry and Forestry Resources Norwegian Institute of Bioeconomy Research Tingvoll Norway; ^4^ Norwegian Institute for Nature Research Trondheim Norway

**Keywords:** *Alces alces*, camera collar, chronic wasting disease, coprophagy, foraging, moose

## Abstract

Coprophagy, the eating of feces, has been documented in a wide range of species but appears to be rare or difficult to detect in deer (Cervidae). Here, we report the first observation of coprophagy in moose *Alces alces*, which was recorded using camera collars on free‐ranging moose in Norway. The footage shows an instance of allocoprophagy by an adult female moose in spring (May). We summarize the current knowledge about coprophagy in deer and briefly discuss potential drivers and possible implications for disease transmission. Further research is needed to determine whether coprophagy occurs frequently in moose and whether this behavior is positive (e.g., increased intake of nutrients) or negative (increased infection by parasites or pathogens).

## Graphical abstract

We report the first observation of coprophagy in moose, which was recorded using camera collars on free‐ranging moose in Norway, and briefly summarize the state of knowledge of this novel behavior in other deer species. Further research is needed to determine whether coprophagy occurs frequently in moose and whether this behavior is positive (e.g., increased intake of nutrients) or negative (increased infection by parasites or pathogens).

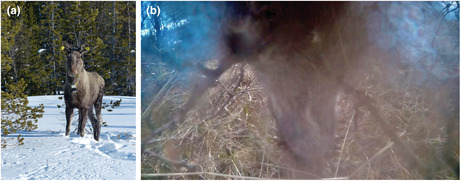

## 1 INTRODUCTION

The term ‘coprophagy’ refers to the ingestion of feces from various sources such as an animal's own (autocoprophagy), those of conspecifics (allocoprophagy), or feces deposited by different species (interspecific coprophagy) (Soave & Brand, 1991).

Coprophagy is well‐known in leporids (Hirakawa, 2001), but the practice has also been observed in a wide range of other organisms, from insects (Körner et al., 2016), fish (Rempel et al., 2022), rodents (Kenagy & Hoyt, 1979), and canids (Waggershauser et al., 2022), to large herbivores such as African elephant (Leggett, 2004), and non‐human primates (Krief et al., 2004).

**FIGURE 1:**
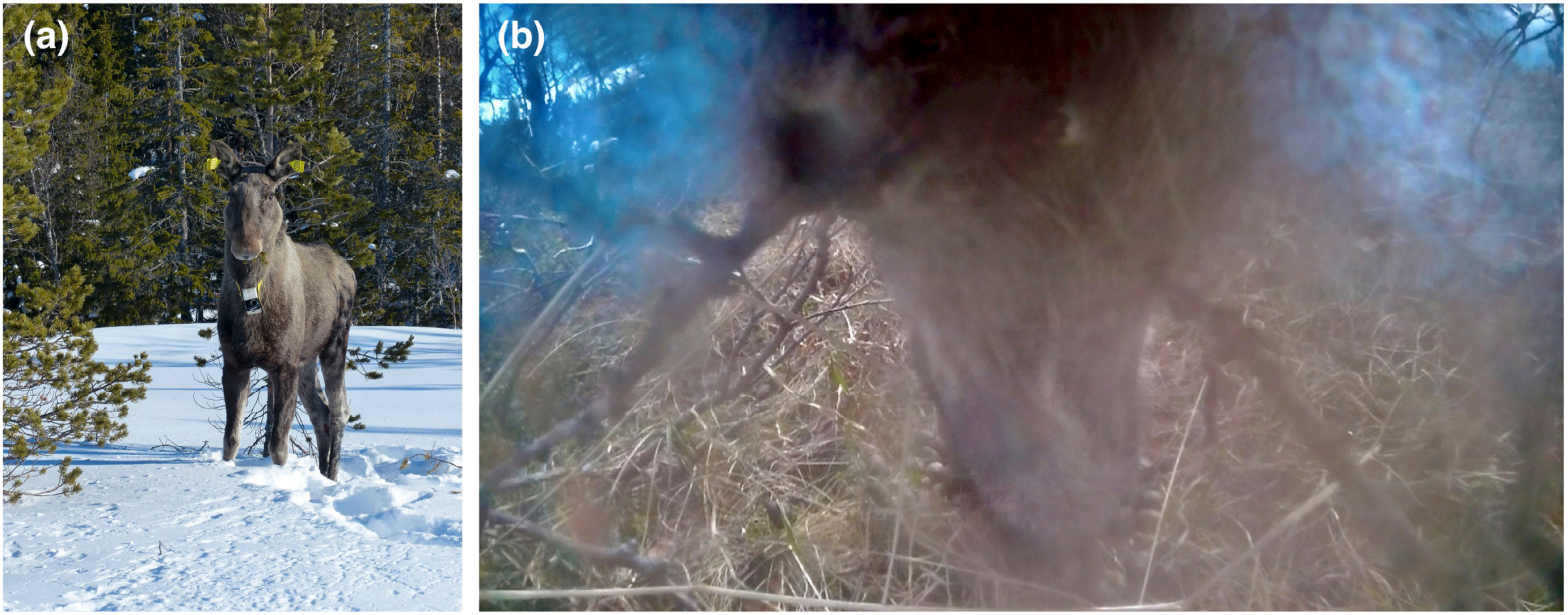
Female moose with a camera collar (a) and video still showing coprophagy by a female moose during spring (May) in the Norwegian county of Trøndelag (b). The full video is available at https://doi.org/10.5061/dryad.t76hdr84j

In deer (Cervidae), coprophagy has only been reported as interspecific coprophagy and for three species: sika deer *Cervus nippon yakushimae* eating the feces of Japanese macaques (Nishikawa & Mochida, 2010), Indian Muntjac *Muntiacus vaginalis* feeding on Asian elephant dung (Ranade & Prakash, 2015), and reindeer *Rangifer tarandus platyrhynchus* ingesting goose droppings (van der Wal & Loonen, 1998). This suggests that the behavior may indeed be rare or, alternatively, difficult to detect as this requires close direct observation of foraging behavior. Here, we report an observation of allocoprophagy in moose *Alces alces* based on video footage that was captured with a camera collar on a wild adult moose cow in Norway (Figure 1). To the best of our knowledge, this constitutes the first observation of any form of coprophagy in moose.

Coprophagy and environmental contamination with feces have been implicated as possible routes for the horizontal transmission of chronic wasting disease (CWD), a fatal prion disease that affects wild and captive cervids such as deer and moose (Miller et al., 2004; Miller & Williams, 2003; Safar et al., 2008). In Norway, CWD was for the first time detected in 2016 in reindeer (Benestad et al., 2016) and moose (Pirisinu et al., 2018), leading to the extensive expansion of CWD surveillance in Norwegian cervid populations (Rolandsen et al., 2022). The latter included the deployment of GPS collars on a large number of moose, red deer *Cervus elaphus*, and reindeer in the affected areas in order to study patterns of space use and how these may affect risks of transmission and disease spread (C.M. Rolandsen, unpublished data). Therefore, a better understanding of why and to what extent coprophagy occurs in wild cervids may also improve our understanding of disease transmission routes.

## 2 MATERIALS AND METHODS

As part of a study of moose behavior and spatial dynamics, five free‐ranging female adult moose were outfitted with camera collars (VERTEX Plus; Vectronic Aerospace GmbH) in the Norwegian counties of Finnmark (three females; N 70°, E 29°) and Trøndelag (one female and one male; N 64.9°, E 11.5°). The county of Trøndelag is where the first two CWD‐positive moose were detected in 2016 (Pirisinu et al., 2018). Automated video sequences (20 s in Finnmark and 30 s in Trøndelag) were recorded daily (8/day in Finnmark and 5–8/day in Trøndelag) from May to September 2017 (Finnmark) and March 2018 to February 2019 (Trøndelag). The recording schedule was preprogrammed to account for the changing length of daylight throughout the study period because the cameras did not have a night‐vision mode. All collaring of study animals was conducted in accordance with standard procedures by approved field personnel, after permits were granted by the Norwegian Animal Research Authority (Finnmark; case no 2015/225449, Trøndelag; case no 16/258650) and the Norwegian Environment Agency. At the end of the study, the camera collars were released remotely, and the video footage was downloaded and visually interpreted. To scan the literature for previous records of coprophagy in moose, we searched the Web of Science and Scopus databases on 14 September 2022 and 3 January 2023 using the Boolean search terms: coprophag* (Topic) AND (deer OR cervidae OR moose Or alces) (Topic), and manually screened the title, abstract, and keywords of the search hits.

## 3 RESULTS

In total, 6504 video sequences were recovered from the five camera collars. Foraging accounted for 24%–38% of the observation time across the five individuals ($\bar{x}$ = 31.3%). Coprophagy was observed only once, in a female moose on the 20 May 2018 at 04:30 PM in the county of Trøndelag (63°15.942′N, 11°51.273′E). This female was estimated to be 3–4 years old based on tooth wear of the front teeth when marked. The 30‐s video recording (https://doi.org/10.5061/dryad.t76hdr84j) shows the individual first briefly feeding on a mountain birch *Betula pubescens* shrub, then searching the nearby ground before homing‐in on and ingesting fresh moose fecal pellets. The literature search resulted in 43 search hits in the Web of Science and 7 search hits in Scopus. Only three studies (cited in the introduction) yielded records of coprophagy in cervids. No study reported coprophagy in moose.

## 4 DISCUSSION

The fact that we observed only one instance of coprophagy during several months of observation suggests that this behavior is a rare occurrence in moose. However, the total recording time per day was only 4 min (8 × 30 s), which corresponds to merely 0.28% of a 24 h period. Moreover, our observations were largely confined to daylight hours, whereas moose are also active at night (Dussault et al., 2004). In consequence, truly rare behaviors would have been likely to escape detection altogether. Thus, even one detection of coprophagy could indicate that the practice may be more common than a single observation would suggest.

Further observations are required to establish whether coprophagy does in fact constitute a causally driven behavior in moose or whether our observation corresponded to an isolated incident, motivated perhaps simply by curiosity or being an inadvertent by‐product of foraging on the ground.

It is difficult to speculate on potential drivers for allocoprophagy in ruminants like moose. Coprophagy may be of nutritional significance by providing an additional source of energy, nutrients, and minerals, especially to newborn animals (Aviles‐Rosa et al., 2019; Körner et al., 2016; Soave & Brand, 1991). Another possibility is that gut microflora or components needed for gut immunocompetence are being transferred to offspring via maternal feces as has been suggested for domestic horses (Beaver, 2019). In central Norway, the month of May is still early in the vegetation period and fresh forage is in short supply. It also marks the beginning of the calving period for moose in our study area. Under such circumstances, coprophagy may conceivably aid female moose in addressing potential imbalances or shortages of minerals in their diet. Another possibility is that the deposition of feces has a fertilizing effect on the surrounding vegetation (Hobbs, 1996), thereby increasing its attractiveness to foraging herbivores.

Like the direct consumption of feces, ingestion of nearby vegetation could have implications for the horizontal transfer of CWD as has been suggested for mule deer feeding near infected carcasses where the flush of nutrients had produced lush vegetation (Miller et al., 2004). However, based on the current understanding of the various types of CWD in Norway (Mysterud et al., 2021; Nonno et al., 2020), horizontal transmission by coprophagy is mainly relevant for animals infected with ‘classical’ contagious CWD, and not the novel, likely sporadic types of CWD found in Norwegian moose (Pirisinu et al., 2018) and red deer (Vikøren et al., 2019). This is because prions are restricted to the central nervous system for sporadic prion diseases, while for the contagious types, animals shed prions in body fluids and excreta. Understanding all possible transmission routes, and identifying those that potentially can be controlled, is of high priority for both types of CWD (Mysterud et al., 2021; Tranulis et al., 2021).

In this context, the Norwegian authorities have banned supplemental feeding and artificial saltlicks targeting cervids to reduce contact rate and accumulation of prions in the environment due to aggregations of animals (Mysterud et al., 2019). However, it is difficult to see a practical way to mitigate transmission by coprophagy in natural settings, except by reducing host density. It has been suggested that animals avoid parasites and diseases by avoiding infectious agents, e.g., in feces (Curtis, 2014; Weinstein et al., 2018). However, as our data suggest, moose do not always do so, whether on purpose or not.

Despite some technical limitations such as storage capacity for recordings and battery life, camera collars facilitate direct, close‐up observations of their carriers and may therefore be a suitable method to further investigate coprophagy and foraging in moose and other ungulates.

## AUTHOR CONTRIBUTIONS

**Robert Spitzer:** Conceptualization (equal); data curation (supporting); formal analysis (equal); methodology (equal); writing – original draft (lead). **Cecilia Åström:** Conceptualization (supporting); data curation (equal); formal analysis (supporting); methodology (supporting); writing – original draft (supporting). **Annika Felton:** Conceptualization (equal); data curation (supporting); formal analysis (supporting); methodology (equal); writing – original draft (equal). **Monica Eriksson:** Conceptualization (supporting); data curation (supporting); formal analysis (supporting); methodology (supporting); writing – original draft (supporting). **Erling L. Meisingset:** Conceptualization (equal); data curation (supporting); formal analysis (equal); funding acquisition (equal); methodology (equal); writing – original draft (equal). **Erling J. Solberg:** Conceptualization (equal); data curation (supporting); formal analysis (equal); funding acquisition (equal); methodology (equal); writing – original draft (equal). **Christer M. Rolandsen:** Conceptualization (equal); data curation (supporting); formal analysis (equal); funding acquisition (lead); methodology (equal); writing – original draft (equal).

## CONFLICT OF INTEREST

The authors have no conflicts or competing interests to declare which are relevant to the content of this article.

## Data Availability

Camera collar recording of allocoprophagy by a female moose, 20 May 2018 at 04:30 PM, Norway (63°15.942′N, 11°51.273′E), MPEG4 video (30 s). Available at https://doi.org/10.5061/dryad.t76hdr84j.
